# OA08 Atypical femoral fracture secondary to long term bisphosphonate use; are we doing enough to prevent it?

**DOI:** 10.1093/rap/rkad070.008

**Published:** 2023-09-27

**Authors:** Arjun Raj, Veena Patel, Preethu Anand

**Affiliations:** University Hospital of Leicester, Leicester, United Kingdom; Rheumatology department at University Hospital of Leicester, Leicester, United Kingdom; University Hospital of Leicester, Leicester, United Kingdom

## Abstract

**Introduction:**

Atypical femoral fractures (AFF) are low energy subtrochanteric or femoral shaft fracture associated with long term use of bisphosphonates (BP). Although the absolute risk of AFF is low and ranges between 3.2-50 cases/100,000 person-years, this risk is said to double with prolonged use (>5 years). ROS and American Society for Bone and Mineral Research (ASBMR) recommends regular review of patient at 5 years for oral BP and 3 years for intravenous BP. We would like to share our experience with two AFF which was seen in our bone clinic over the last one year resulting from prolonged bisphosphonates use.

**Case description:**

Case 1

A 76-year-old Caucasian lady was reviewed for follow-up appointment in bone clinic for the management of steroid induced osteoporosis. She was on AA for 13 years for primary prophylaxis. It was noted that she was experiencing discomfort in her right thigh for the last 8 months. Her initial femoral X-ray showed incomplete AFF of the right femur and she was referred to orthopaedics who suggested conservative management. 6 months later, she unfortunately had a fall resulting in right femur fracture which required intramedullary nailing. X-ray of the contralateral side shows periosteal thickening and she is waiting for prophylactic nailing.

Case 2

79 year old white Caucasian presented to the hospital with a fall. She has been on long term steroids for PMR and was on alendronate for 20 years for primary prophylaxis. X-ray showed complete AFF of proximal right femur shaft which required intramedullary nailing, subsequently developed pain in left hip during physiotherapy and X-ray showed incomplete AFF of mid left femur which needed prophylactic nailing. She was then referred to our clinic for further risk stratification.

Both the patients had their bisphosphonates stopped and was advised reduced weight bearing along with calcium and vitamin D supplementation.

The table summarises the background history and investigations post presentation with AFF:

**Discussion:**

Bisphosphonates are one of the mainstays for managing steroid induced osteoporosis and for patients with high risk fragility fractures. Despite the increasing awareness of long term complication, we still see patients with AFF who have missed the opportunity for review in primary and secondary care particularly among patients with primary prophylaxis with satisfactory DEXA scan.

ROS and ASBMR recommends review of patients who are on oral bisphosphonates at 5 years and for patients on intravenous bisphosphonates at 3 years for further risk stratification to reduce the incidence of AFFs. It also recommends close monitoring in patients with prodromal pain in the hip, groin or thigh especially in those who have high risk factors such as long term glucocorticoid use, Asian race and femoral bowing. Cessation of bisphosphonates can result in 70% reduction of developing AFF. Hence a break in the treatment for approximately 18-24 months needs to be considered for patients who are at lower levels of risk for fragility fracture such as age <75years, no previous history of fragility fracture, DEXA T score more than −2.5 and low FRAX score. Yet, this is not happening in routine clinical practice both in primary and secondary care and patients fall out of safety netting resulting in AFF. We need robust pathway and safety netting for patients who are on long term treatment.

This situation could have also been exacerbated by the COVID pandemic which has increased the waiting lists and cancelled appointments. Resulting in many elderly patients having no follow ups and DEXA assessments for risk stratification.

**Key learning points:**

Following the COVID pandemic there has been a huge impact on the waiting list, which has further pushed follow-up review for patients on long term bisphosphonates. This has resulted in a missed opportunity to identify patients with low risk to have a break in the BPs treatment. We feel that with appropriate treatment pathways needs to be set at primary care and secondary care level to capture these patients on long bisphosphonates after 3 to 5 years of BP use; especially patients who are at higher AFF risk, including patients on glucocorticoids and patients of Asian ancestry.

Steps must be put in place to ensure counselling and education is given to patients about risk of AFF and prodromal symptoms such as thigh or groin pain which are initial symptoms, so that they can seek appropriate review at primary care level.

ROS and ASBMR Task Force 2013 need to be implemented locally to capture patients with long term BPs treatment. Also, if patients has AFF fracture, imaging of the other leg has to be considered for further evaluation as more than one fourth of the cases have contralateral lesion.

**Table:** OA08 Table

Case	Past medical history	Medication	DEXA	Vitamin D mmol/L	Procollagen type 1 peptide ug/L	Beta-C-terminal telopeptide ug/L
1	Waldenstrom macroglobulinemia Steroid-induced osteoporosis Splenectomy	Vitamin D Omeprazole Azothioprine Prednisolone	T-score at the lumbar spine −0.1 Left and right neck of the femur −0.2	46	19	
2	Asthma Non-epileptic seizures Polymyalgia rheumatica	Prednisolone Ropinirole Salbutamol inhaler Lansoprazole Lamotrigine	T-score Lumbar spine 1.1	55	14	0.14

